# Topology Optimization of Passive Micromixers Based on Lagrangian Mapping Method

**DOI:** 10.3390/mi9030137

**Published:** 2018-03-20

**Authors:** Yuchen Guo, Yifan Xu, Yongbo Deng, Zhenyu Liu

**Affiliations:** 1 Changchun Institute of Optics, Fine Mechanics and Physics (CIOMP), Chinese Academy of Science, Changchun 130033, China; guoyuchen15@mails.ucas.edu.cn; 2 University of Chinese Academy of Science, Beijing 100049, China; 3 State Key Laboratory of Mechanical System and Vibration, Shanghai Jiao Tong University, Shanghai 200240, China; xuyifan19890219@sjtu.edu.cn; 4 State Key Laboratory of Applied Optics, Changchun Institute of Optics, Fine Mechanics and Physics (CIOMP), Chinese Academy of Science, Changchun 130033, China; dengyb@ciomp.ac.cn

**Keywords:** passive micromixer, topology optimization, Lagrangian description, mapping method

## Abstract

This paper presents an optimization-based design method of passive micromixers for immiscible fluids, which means that the Peclet number infinitely large. Based on topology optimization method, an optimization model is constructed to find the optimal layout of the passive micromixers. Being different from the topology optimization methods with Eulerian description of the convection-diffusion dynamics, this proposed method considers the extreme case, where the mixing is dominated completely by the convection with negligible diffusion. In this method, the mixing dynamics is modeled by the mapping method, a Lagrangian description that can deal with the case with convection-dominance. Several numerical examples have been presented to demonstrate the validity of the proposed method.

## 1. Introduction

Lab-on-a-chip devices have been widely used in the area of the analysis, synthesis, and separations due to the advantages of high efficiency, portability, and low reagent consumption [1]. Injection, mixing, reaction, cleaning, separation, and detection, which are the functions of conventional analytical laboratory, can be achieved on a centimeter-level chip [2]. Various microfluidic devices have been integrated in lab-on-a-chip, such as micropumps, microvalves, micromixers, and microchannel. Rapid and complete mixing can influence the efficiency of a microfluidic system [3]. Therefore, micromixer plays a significant role in the lab-on-a-chip devices. Based on actuation methods, micromixers can be classified into two categories: active micromixers and passive micromixers [4]. Active mixers use external energy to create chaotic convection, such as pressure [5], magnetohydrodynamics [6], electrokinetics [7] and acoustics [8,9,10]. Active mixers have short mixing times and distances and can be controlled to be on and off, according to the needs of the users. Because of the requirement of external energy, however, the fabrication and integration of active mixers is complicated and expensive [11]. Comparatively, passive mixers can achieve fluid mixing solely by the geometries of channels and be integrated in a complex microfluidic system simply and directly. The inconsistent cross sections of a microchannel can cause the fluid to be stretched and folded in the transversal direction [12,13,14,15]. A reasonable layout of passive micromixer can strengthen the chaotic convection to enhance the mixing performance. Immiscible fluids are widely used in chemical industry, where the particle flow can be considered as the immiscible fluids [16]. The mixture of immiscible fluids appears in biochemical experiments, which is usually not discussed compare to the mixers with phenomenon of convection-diffusion. With the trend of miniaturization in recent years, the micromixers of immiscible fluids with efficient mixing performance are also desired in a microfluidic system.

Topology optimization of fluid flows has been proposed by Borrvall and Petersson for Stokes flow [17] and the Navier-Stokes flow [18,19,20,21]. When compared to shape optimization method, the detail topology and shape of the microchannels can be obtained simultaneously by topology optimization. This method has been used to design the microchannel networks, micropumps, no-moving part microvalves, and micromixers [22,23,24,25]. In the topology optimization of micromixers, the convection-diffusion equation is usually used to describe the mixing process of the fluids, and objective function is the variance between the actual obtained concentration at the outlet and the expected concentration. All of the topological optimization methods that are mentioned above used to design the mixers of miscible fluids are based on the Eulerian description of the convection-diffusion dynamics and can not be directly applied to the design of immiscible fluid mixers. The mixture of immiscible fluids is a convective problem in physics and should be described by the Navier–Stokes (NS) equations and convection equation in numerical calculation. However, when using the standard Galerkin finite element method to solve the above equation, the numerical instability will occur. For an optimization process, the numerical instability of solving forward problems is a big challenge. To avoid this problem, the mapping method is used in this paper to describe the convection problem in numerical calculation. The mapping method proposed by Singh et al. [26,27] can describe the mixing performance by calculating the mapping matrix and be integrated into the topology optimization method. For the topology optimization problem, the measure of the mixing in the mapping method needs to be discussed. The coarse grained concentration [28] can change the value of the objective function by replacing the concentration in the objective function with the coarse grained concentration defined in discrete areas. In order to shorten the optimization time, the mixing performance of entire periodic structure is obtained by analyzing only one cycle structure in this paper.

In passive mixers, the mixing efficiency is mainly determined by the layout of mixers. The chaotic convection can be promoted by the various cross-sectional structures along the flow direction. Based on the topology optimization method of fluidic flows and Lagrangian description, this paper is focused on the layout design method of the passive micromixers of immiscible fluid, where the mapping method is used to describe the mixing process of the immiscible fluid. A new mixing measurement is applied in this paper, based on the change of position of the mixed fluids. This paper is organized as follows: the choice of mixing measurement and the mapping method are stated in Section 2; the topology optimization model of the passive micromixers and the corresponding adjoint equations and derivative are derived in Section 3; several numerical results are presented in Section 4; and, the discussion and conclusion are stated in Section 5.

## 2. Measure of Mixing and Lagrangian Mapping Description

### 2.1. Measure of Mixing

In the topology optimization method of micromixers based on Eulerian description of the convection-diffusion dynamics, we define the concentration as the volume fraction of a fluid in a mixture of two fluids at a point. To quantify and compare mixing performance, the least squares variance between the actual obtained concentration at the outlet and the expected concentration can be expressed as [29]:

(1) $$J(c)=\frac{\int_{\Gamma_{out}}{\left(c-\bar{c}\right)}^{2}d\Gamma }{\int_{\Gamma_{in}}{\left(c_{0}-\bar{c}\right)}^{2}d\Gamma }$$

where Г*_in_* and Г*_out_* are the inlet and outlet of the mixer, respectively; *c*_0_ is the reference concentration, which is usually the designer specified concentration at the inlet. Ideally, the two fluids are sufficiently mixed in the mixer. Therefore, the expected concentration $\bar{c}$ is chosen as the ideal concentration after sufficient mixing at the outlet, which is the average concentration. The concentrations of two fluids are set to be dimensionless value 0 and 1. When the two fluids are miscible, *J*(*c*) = 0 means that the two fluids can be considered to be completely mixed and *J*(*c*) = 1 means that the two fluids can be considered to be completely separated. However, when the two liquids are immiscible, the concentration c of a fluid is always be 0 or 1, due to the absence of diffusion and *J*(*c*) remains constant. The expression in Equation (1) is not suitable to quantify the mixing performance. The coarse grain concentration method that was proposed by Welander et al. [28] can avoid this situation. The coarse grain concentration *C_i_* defined on a finite cell Г*_i_* is:

(2) $$C_{i}=\int_{\Gamma_{i}}c(X)d\Gamma$$

where Г*_i_* is the *i*-th cell obtained discretely on the cross section perpendicular to the flow direction; *C_i_* can vary in interval [0,1]. Therefore, Equation (1) can be rewrite as

(3) $$J_{\bar{C}}(C)=\frac{1}{A_{\Gamma }}\sum_{i=1}^{N}\frac{{\left(C_{i}-\bar{C}\right)}^{2}}{{\left(C_{i}^{0}-\bar{C}\right)}^{2}}A_{\Gamma_{i}},\bar{C}=\frac{1}{A_{\Gamma }}\sum_{i=1}^{N}C_{i}A_{\Gamma_{i}}$$

where $A_{\Gamma }$ and $A_{\Gamma_{i}}$ is the area of cross section and the *i*-th cell, respectively; $\bar{C}$ is the average coarse grain concentration; $C_{i}^{0}$ is the coarse grain concentration of the *i*-th cell on the cross section of inlet; and *N* is the number of cells divided in cross section.

Since the mixed fluids are immiscible, the coarse grain concentration changes only at the contact cells of the two fluids. Due to the low Reynolds numbers of fluid and the limited mixing length of micromixer used in the topology optimization model, the change of the value of mixing performance in Equation (3) is not large enough. When the number of discrete cells increases at the same time, the value of Equation (3) is not sensitive to the change of coarse grain concentration. A new measurement mixing is proposed to amplify the value modification in the mixing performance by using the change of the positions of two fluids at the cross section of outlet. The least squares variance between the actual obtained coarse grain concentration *C_i_* at the outlet and the initial coarse grain concentration $C_{i}^{0}$ at the inlet is used:

(4) $$J_{C^{0}}(C)=\frac{1}{1+\frac{1}{A_{\Gamma }}\sum_{i=1}^{N}{\left(C_{i}-C_{i}^{0}\right)}^{2}A_{\Gamma_{i}}}$$

When $J_{C^{0}}(C)=1$, the two immiscible fluids can be considered to be completely separated; when $J_{C^{0}}(C)<1$, the two immiscible fluids can be considered to be mixing.

### 2.2. Mapping Method

A distribution matrix is used in the mapping method to store the information, which describes the changes of fluidic distribution between two specified cross sections [30,31]. To obtain the each coefficient of mapping matrix, the initial cross section is divided into a large number of discrete cells with specified size. The material of fluid transferred to several recipient cells from a donor cell along the fluidic flow. The fraction of material in the recipient cell Ω*_j_* in section at *X* = *X*_0_ + Δ*X*, which is found in the donor cell Ω*_i_* in section at *X* = *X*_0_ is the coefficient of mapping matrix. The cross section with *N* cells can construct a distribution matrix of the order *N* × *N*:

(5) $$\varphi_{ij}=\frac{\int_{\Omega_{j}|{}_{x=x_{0}+\Delta x}\cap \Omega_{i}|{}_{x=x_{0}}}dA}{\int_{\Omega_{j}|{}_{x=x_{0}}}dA}$$

To describe the detail of flow, a lot of sections are traced which are set along the flow direction. Tracing all cells in all sections during a flow over a distance Δ*X*, the detail of flow can be described by the complex deformation of cells. Although this tracking method is feasible, it is too time-consuming to apply into topology optimization. Singh et al. proposed a convenient method for calculating the coefficients of the mapping matrix [26,27]. To approximate the coefficients of mapping matrix, K markers are filled into each cell uniformly in donor section at *X* = *X*_0_, and then, tracing these markers can obtain the information about the distribution in recipient section at *X* = *X*_0_ + Δ*X*. When the number of markers in the cell Ω*_j_* in donor section is *M_j_* and *M_ij_* markers are traced in the cell Ω*_i_* in recipient section, the coefficient of mapping matrix can be calculated as

(6) $$\Phi_{ij}=\frac{M_{ij}}{M_{j}}$$

The convection dynamics of fluids can be analyzed the following procedure. Using the same cell to describe the coarse grain concentration, the coarse grain concentration distribution of cross-section can construct a vector $C\in R^{N\times 1}$ (*N* is the number of cells). After passing through the structure that is described by the mapping method, the coarse grain concentration distribution of recipient section C*^r^* can be calculated from the coarse grain concentration distribution of donor section C*^d^* as:

(7) $$C^{r}=\Phi C^{d}$$

The coarse grain concentration distribution at outlet can be obtained as:

(8) $$C_{o}=\Phi_{all}C_{i}$$

where C*_i_* and C*_o_* are the coarse grain concentration distribution of inlet and outlet cross-section, respectively; and Φ*_all_* is the mapping matrix of whole mixer.

Since the construction of the mapping matrix has no correlation with the initial cross-section concentration distribution, the mixer of periodic layout, with a known initial mapping matrix Φ^1^ of a single cycle, has the following relation:

(9) $$C^{i+1}=\Phi^{1}C^{i},C^{n}=\underset{n\;\mathrm{times}}{\underset{︸}{(\Phi^{1}(\Phi^{1}(\ldots (\Phi^{1}}}C^{0}))))$$

where C*^i^* is the coarse grain concentration distribution after the *i*-th mixing. Therefore, for the periodic mixers in any cycle, the computational cost is saved that the concentration distribution. C*^n^* can be obtained by simply multiplying the single cycle matrix in corresponding times.

**Figure 1:**
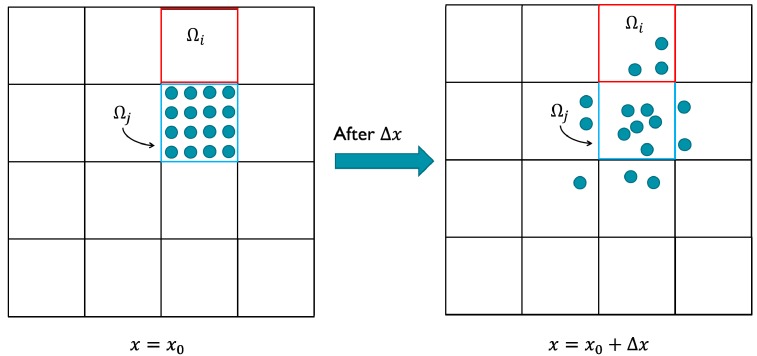
The calculation of the mapping coefficient Φ*_ij_* in the mapping matrix is the ratio of the number of markers received by the recipient cell Ω*_i_* at *X* = *X*_0_ + Δ*X* to the initial number of markers in Ω*_j_* at *X* = *X*_0_ (in this example Φ*_ij_ =* 3 /16) [26,27].

Assuming that the length direction of mixing channel is the *x*-axis, the cross section that is shown in Figure 1 is the *y*-*z* section. The trajectories of markers are tracked based on the coordinate axis. Tracing can be realized by the axial velocity component u*_x_* and the transversal velocity components u*_y_* and u*_z_*, respectively:

(10) $$\frac{dy}{dx}=\frac{u_{y}}{u_{x}},\frac{dz}{dx}=\frac{u_{z}}{u_{x}}$$

Since the particles become disordered at any downstream position in forward tracing method, there is no guarantee that an equal number of traced markers can be found in each cell at the outlet. Using the backward particle tracing (BTP) to construct the distribution matrix, the markers initially fill the recipient cell in the cross section of outlet, and they are traced backward against the flow direction [26,27],

(11) $$X_{i}=X_{0}+\sum_{i=1}^{n}\left(\int_{x_{i}+\Delta x}^{x_{i}}\frac{u}{u^{T}I_{u}}dX\right)$$

where *X_i_* = (*x_i_*, *y_i_*, *z_i_*) and *X*_0_ = (*x*_0_, *y*_0_, *z*_0_) are the coordinates of the markers at the cross section of inlet and outlet, respectively; $I_{u}={\left[1\;0\;0\right]}^{T}$; *n* = *n_all_* − 1, *n_all_* is the number of cross section in the mapping method. Due to the integration of axial spatial increment rather than time, the error that is caused by the different time distribution can be eliminated. However, this approach is not effective when the fluid is reflowing.

**Figure 2:**
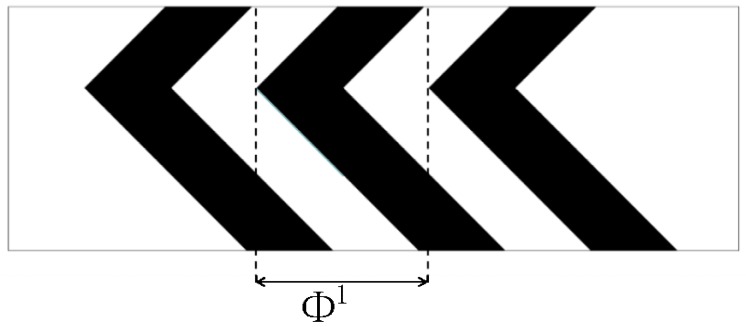
Schematic representation of the grooves in a staggered herringbone mixer (SHM). The mapping matrix Φ^1^ covers a single groove applying a fully developed velocity field.

**Figure 3:**
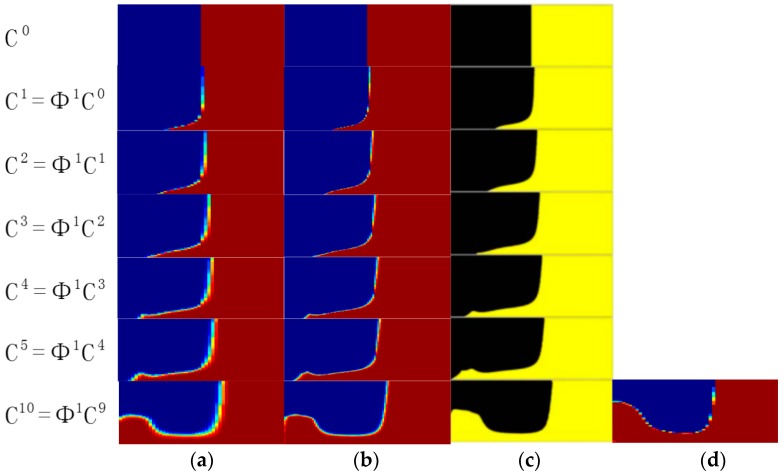
The evolution of coarse grain concentration distribution C*^i^* in a SHM with one groove type: (**a**) The result of mapping method which the tracing cross section is covered with 50 × 50 cells, and each cell is filled with 100 uniformly distributed markers; (**b**) The result of mapping method which the tracing cross section is covered with 100 × 100 cells, and each cell is filled with 100 uniformly distributed markers; (**c**) The results in the study of Singh et al. which the tracing cross section is covered with 200 × 200 cells, and each cell is filled with 256 uniformly distributed markers [26]; (**d**) The coarse grain concentration distribution at outlet of SHM with ten grooves.

Figure 2 shows a top view of the grooves on the bottom in a staggered herringbone mixer (SHM). We apply the geometry of SHM and the material properties used in the study of Singh et al. [26]. The length ratio of two arms of every groove is 2:1, and all arms are at 45° to the axial direction; the channel height *h* = 77 μm, the channel width *w* = 200 μm, the depth of grooves *g_d_* = 17.7 μm, the width of grooves *g_w_* = 70.7 μm and the distance between two grooves also equals 70.7 μm; the viscosity and density of the fluid in the micromixers are 0.067 kg·m/s and 1.2 × 10^3^ kg/m^3^. The average inlet velocity *u* = 0.2 cm/s. The velocity field is obtained by 144,000 hexahedral elements and 155,031 nodes. Figure 3a–c show the mixing evolutions in a SHM consisting of one groove type, whose mapping matrix is represented by Φ^1^. In the mapping computations of Figure 3a,b, the tracing cross section is covered with 50 × 50 and 100 × 100 cells, respectively, and each cell is filled with 100 uniformly distributed markers. Figure 3c shows the results in the study of Singh et al. [26] using the above parameters. Figure 3d shows the coarse grain concentration distribution at outlet of SHM with ten grooves. The results in Figure 3 demonstrated the validity of the mapping method that is used in this paper. These parameters of mapping method will be used in the optimization process.

## 3. Topology Optimization Model of Mixers

When the area of each cell divided by the mapping method is the same, the Equation (4) can be simplified to

(12) $$J_{C^{0}}(C)=\frac{N}{N+\sum_{i=1}^{N}{\left(C_{i}-C_{i}^{0}\right)}^{2}}$$

where *N* is the number of cells divided in cross section. This discrete equation is used as quantitative criterion to measure the mixing performance of the immiscible fluids. In this paper, the key point is how to find a reasonable layout of a micromixer in a designer specified design domain, which minimizes the quantitative criterion and mixing can be obtained using the topology optimization method. Based on the continuity assumption, the fluidic field will be described by the Navier-Stokes equations:

(13) $$\begin{array}{c} \rho (u\cdot \nabla )u-\eta \Delta u+\nabla p=f\\ -\nabla \cdot u=0 \end{array}$$

where u, *p*, *ρ*, and *η* are the velocity, pressure, density, and viscosity of the fluid, respectively; f is the body forces acting on the fluid. In the topology optimization method, an artificial friction force is introduced into the Navier-Stokes equations, which was proposed for the Stokes flow by Borrvall and Petersson [17] and generalized to the Navier-Stokes flow by Gersborg et al. [20] and Olesen et al. [21]. Initially, an artificial porous material is uniformly distributed in the design domain; and then, the artificial porous material forms solid and liquid phases gradually; at last, the high and low impermeability characterize the solid phase and the fluid phase, respectively. The artificial friction force is f = −*α*(*γ*)u, and *α* is the impermeability of the artificial porous material, and *γ* is the design variable. The design variable *γ* varies in interval [0,1], where 0 and 1 denote the solid and fluid phases, respectively. The impermeability of porous material *α* is the interpolation function of design variable *γ* [17,18,19,20,21,22]:

(14) $$\alpha (\gamma )=\alpha_{\min}+(\alpha_{\max}-\alpha_{\min})\frac{q(1-\gamma )}{\left(q+\gamma \right)}$$

where *α*_max_ and *α*_min_ are the impermeability of the solid phase and the fluid phase, respectively; *q* is a positive value used to adjust the convexity of the interpolation function; *α*_min_ is chosen 0 in the fluidic topology optimization. To obtain the perfect impermeability of solid no-slip boundary, *α*_max_ should be infinite, but a finite number has to be chosen to ensure the numerical stability. The layout of the passive micromixer of immiscible fluid can be determined by seeking the distribution of the design variable *γ*. The topology optimization model based on mapping method is:

(15) $$\begin{array}{l} \min:J_{C^{0}}(C)=\frac{N}{N+\sum_{i=1}^{N}{\left(C_{i}-C_{i}^{0}\right)}^{2}}\\ s.t.\rho (u\cdot \nabla )u-\eta \Delta u+\nabla p=-\alpha u\;\mathrm{in}\;\Omega \\ \;-\nabla \cdot u=0\;\mathrm{in}\;\Omega \\ \;u=u_{0}\;\mathrm{on}\;\Gamma_{in}\\ \;[-pI+\eta (\nabla u+{\left(\nabla u\right)}^{T})]\cdot n=0\;\mathrm{on}\;\Gamma_{out}\\ \;0\leq \gamma \leq 1\\ \;X=X_{0}+\sum_{i=0}^{n}\left(\int_{x_{i}+\Delta x}^{x_{i}}\frac{u}{u^{T}I_{u}}dX\right)\;\mathrm{on}\;\Gamma_{in}\\ \;X=X_{0}\;\mathrm{on}\;\Gamma_{out}\\ \;C=C_{0}\;\mathrm{on}\;\Gamma_{in}\\ \;C=\Phi (y_{i},z_{i})C_{0}\;\mathrm{on}\;\Gamma_{out}\\ \;\Phi_{ij}=\frac{M_{ij}}{M_{j}} \end{array}$$

where $\Omega =\Omega_{D}\cup \Omega_{C}$ is the computational domain; Ω*_D_* is the design domain; Ω*_C_* is the channels connected to the inlet, outlet, and design domain.

The constraint optimization model in Equation (15) can be transferred into unconstrained one by Lagrangian multiplier method:

(16) $$\begin{array}{cc} L= & J+a{\left(u,\lambda_{u}\right)}_{\Omega }+\rho {\left((u\cdot \nabla )u,\lambda_{u}\right)}_{\Omega }+{\left(\nabla p,\lambda_{u}\right)}_{\Omega }+{\left(\alpha u,\lambda_{u}\right)}_{\Omega }\\ & -{\left(g,\lambda_{u}\right)}_{\Gamma_{N}}+{\left(u-u_{0},\lambda_{u}\right)}_{\Gamma_{D}}+{\left(-\nabla \cdot u,\lambda_{p}\right)}_{\Omega }+{\left(C-\Phi (y,z)C_{0},\lambda_{C}\right)}_{\Gamma_{N}}\\ & +{\left(C-C_{0},\lambda_{C}\right)}_{\Gamma_{D}}+{\left(X-X_{0},\lambda_{X}\right)}_{\Gamma_{D}}+{\left(X_{i}-X_{0}-\sum_{i=0}^{n}\left(\int_{x_{i}+\Delta x}^{x_{i}}\frac{u}{u^{T}I_{u}}dX\right),\lambda_{X}\right)}_{\Gamma_{N}} \end{array}$$

where $a{\left(u,\lambda_{u}\right)}_{\Omega }=\int_{\Omega }\eta \nabla u:\nabla \lambda_{u}d\Omega$; ${\left(*,*\right)}_{\Omega }$ and ${\left(*,*\right)}_{\Gamma }$ is the inner product on the computational domain and boundary; *λ*_u_, *λ_p_*, *λ_C_* and *λ_X_* are the adjoint variable of the velocity field, the pressure field, the coarse grain density on the cross outlet section and the coordinate after particle tracing, respectively.

The variation of the Equation (16) is

(17) $$\delta L=\frac{\partial L}{\partial u}\delta u+\frac{\partial L}{\partial p}\delta p+\frac{\partial L}{\partial X}\delta X+\frac{\partial L}{\partial C}\delta C+\frac{\partial L}{\partial \gamma }\delta \gamma$$

According to the Karush-Kuhn-Tucker conditions for the partial differential equation constrained optimization problem, the optimization problem in Equation (15) can be obtained by solving the following adjoint equations [19] (see the Appendix A for the detailed derivation):

(18) $$\left\{ \begin{array}{l} -\eta \Delta \lambda_{u}-\rho (u\cdot \nabla )\lambda_{u}+\rho (\nabla u)\cdot \lambda_{u}+\nabla \lambda_{p}=-\alpha \lambda_{u}\;\mathrm{in}\;\Omega \\ -\nabla \cdot \lambda_{u}=0\;\mathrm{in}\;\Omega \\ \lambda_{u}=0\;\mathrm{on}\;\Gamma_{D}\\ {}[\eta (\nabla \lambda_{u}+{\left(\nabla \lambda_{u}\right)}^{T})-\lambda_{p}I]\cdot n=-\rho (u\cdot n)\lambda_{u}+\sum_{i=0}^{n}\left(\int_{x_{0}+\Delta x}^{x_{0}}\left(\frac{1}{u^{T}I_{u}}I-\frac{u}{{\left(u^{T}I_{u}\right)}^{2}}I_{u}^{T}\right)dX\right)\lambda_{X}\;\mathrm{on}\;\Gamma_{N}\\ \lambda_{X}=\frac{\partial \Phi }{\partial X_{i}}\lambda_{C}\;\mathrm{on}\;\Gamma_{N}\\ \lambda_{X}=0\;\mathrm{on}\;\Gamma_{D}\\ \lambda_{C}=0\;\mathrm{on}\;\Gamma_{D}\\ \lambda_{C}=-\frac{-2N(C-C^{0})}{{\left[N+\sum_{i=1}^{N}{\left(C_{i}-C_{i}^{0}\right)}^{2}\right]}^{2}}\;\mathrm{on}\;\Gamma_{N} \end{array}\right.$$

The adjoint sensitivity of the optimization model in Equation (15) is:

(19) $$\frac{DL}{D\gamma }|{}_{\Omega }=\frac{\partial \alpha }{\partial \gamma }u\cdot \lambda_{u}$$

**Figure 4:**
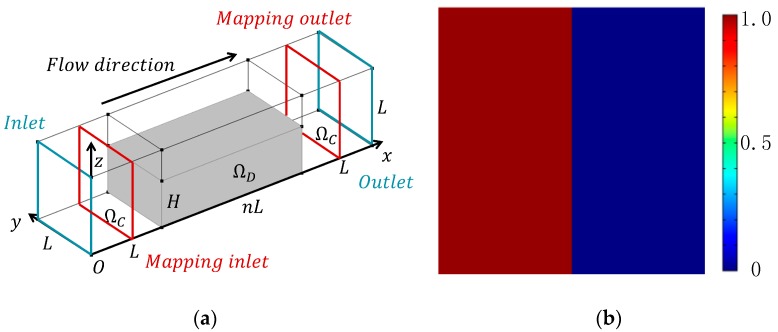
(**a**) Design domain Ω*_D_* of the micromixers at the bottom layer of the straight channel. Ω*_C_* is the channels connected to the inlet, outlet and design domain Ω*_D_*. The length of design domain Ω*_D_* is *n* times of *L*; (**b**) the distribution of coarse grain concentration on the outlet.

When considering the manufacturability, the micromixer is designed to be a single layer structure, like the SHM [12,13,14,32,33]. In the optimization model, an additional constraint is added to ensure that the design variable in the depth direction have a consistent value. The design variable is defined on the plane *x*0*y* (Figure 4), and the adjoint derivative of the optimization model (19) is changed to [22]:

(20) $$\frac{DL}{D\gamma }=\frac{1}{z_{t}-z_{b}}\int_{z_{b}}^{z_{t}}\frac{\partial \alpha }{\partial \gamma }u\cdot \lambda_{u}dz$$

where *z_t_* and *z_b_* are the coordinates in *z* direction of top and bottom surface of the design domain, and *z_t_* > *z_b_*.

The topology optimization problem is solved by using the gradient-based iterative approach. In the optimization iterations, the Navier-Stokes equations, the backward particles tracing equation, and the corresponding adjoint equations in the weak form are solved by the finite element method using the commercial software COMSOL Multiphysics (version 3.5, COMSOL Inc., Stockholm, Sweden). The method of moving asymptotes (MMA) is used to update the design variable. The optimization iterations are stopped, when the maximal change of the objective value in three consecutive iterations less than 1 × 10^−3^. The procedure of solving the topology optimization problem for the layout design of passive micromixers is listed in below.

1. Give the initial value of the design variable *γ*;
2. Solve the Navier-Stokes equations and backward particle tracing equation by the finite element method;
3. Solve the adjoint equation;
4. Compute the adjoint derivative and the corresponding objective and constraint values;
5. Update the design variable by method of moving asymptotes (MMA);
6. Check for convergence; if the stopping conditions are not satisfied, go to 2; and
7. Post-processing

## 4. Results and Discussion

To demonstrate the validity of the proposed method, passive micromixers of immiscible fluid in a straight microchannel with external driven flow (constant flow rate at inlet) is investigated in the following. The design domain is shown in Figure 4a, and each cubic space with an edge size equals to *L* is discretized by 20 × 20 × 20 hexahedral elements. The width of micromixer *L* is 400 μm and the height of design domain *H* is 240 μm. The viscosity and density of the fluid in the micromixers are 1 × 10^−3^ Pa·s and 1 × 10^3^ kg/m^3^. The value of α_max_ and *q* in the topology method are chosen 1 × 10^7^ and 0.1. The initial value of the design variable *γ* is 0.4, which should be between [0,1], as shown in Equation (15). Since the backward particle tracing method is applied, the number of cell in the cross section of outlet is 50 × 50 and 100 markers are traced in each cell. The coarse grain concentrations of two immiscible fluids are set to be dimensionless value 1 and 0 (Figure 4b), respectively. All of the computations are performed on a DELL workstation (DELL Optiplex 7040, Intel Core i7-6700 CPU, 16 gigabyte memory, Round Rock, TX, USA).

**Figure 5:**
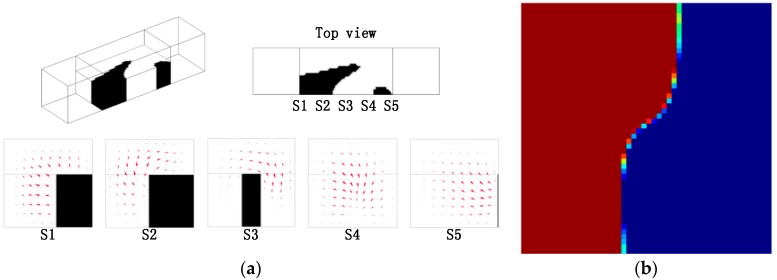
(**a**) Layout of the micromixer for the design domain as shown in Figure 4, where *n* = 2, *Re* = 2.5, and projected velocity vector distribution in the cross sections (S1, S2, S3, S4, S5); the mixing measurement $J_{C_{0}}(C)$ = 0.9141; (**b**) the distribution of coarse grain concentration on the outlet.

**Figure 6:**
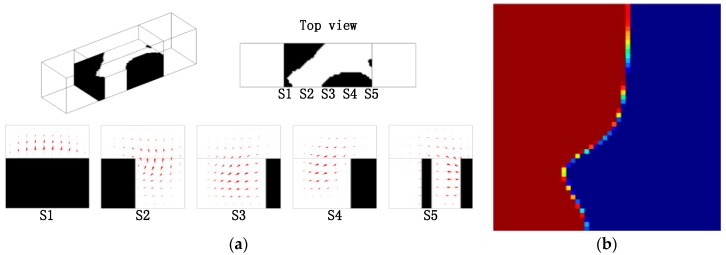
(**a**) Layout of the micromixer for the design domain as shown in Figure 4, where *n* = 2, *Re* = 5, and projected velocity vector distribution in the cross sections (S1, S2, S3, S4, S5); the mixing measurement $J_{C_{0}}(C)$ = 0.9147; (**b**) the distribution of coarse grain concentration on the outlet.

**Figure 7:**
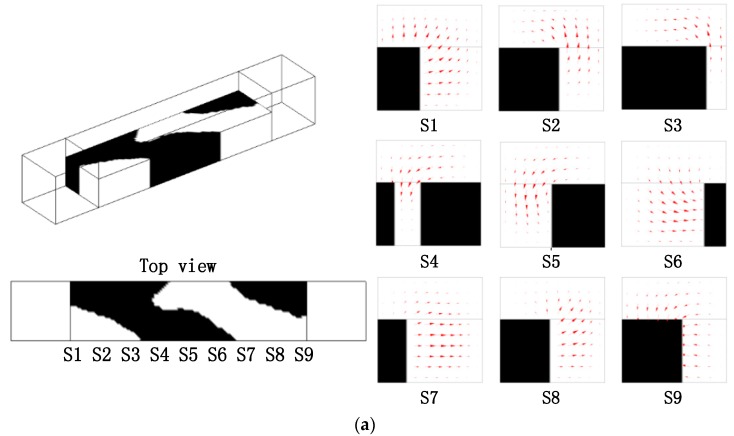

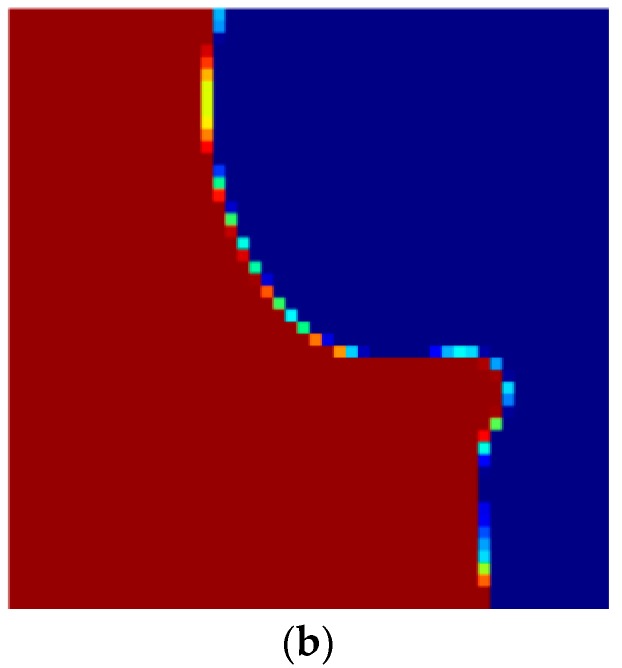
(**a**) Layout of the micromixer for the design domain as shown in Figure 4, where *n* = 4, *Re* = 5, and projected velocity vector distribution in the cross sections (S1, S2, S3, S4, S5, S6, S7, S8, S9); the mixing measurement $J_{C_{0}}(C)$ = 0.8382; (**b**) the distribution of coarse grain concentration on the outlet.

Based on the topology optimization model in Section 3, the optimal layouts of the passive micromixers of immiscible fluid with different Reynolds number and length of the design domain are obtained, as shown in Figure 5, Figure 6 and Figure 7. The optimized results strongly depend on the parameters, such as the size of design domain and the Re number. From the comparison between the results in Figure 5 and Figure 6, the obtained layouts of micromixers depend on the selection of Reynolds number. Different values of n are chosen to compare the mixing performance of the micromixers obtained by the topology optimization method (Figure 6 and Figure 7). A larger value of *n* means a longer mixing length and a more reasonable layout in the micromixers. When the length of the mixer is relatively short, the herringbone type structure is not necessary; when the length of single-periodic becomes longer, the herringbone type structure promotes the mixing effect obviously. By comparing the optimal results, the herringbone type structure is a reasonable topology, which could be used further for detailed shape or size optimization when the mixer has an appropriate length.

**Figure 8:**
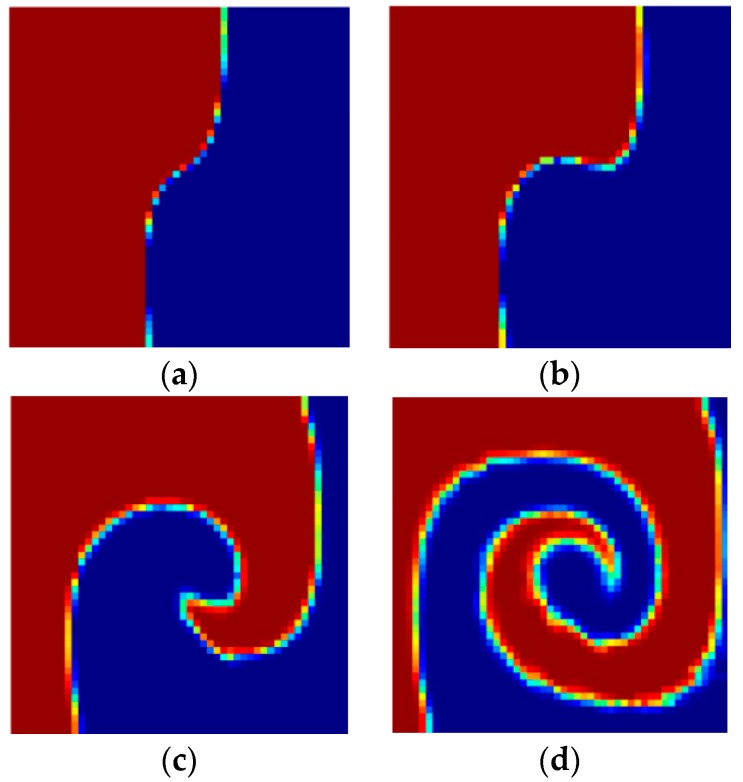
The evolution of mixing performance of micromixers with different cycles which the single cycle is the layout in Figure 5: (**a**–**d**) are the distribution of coarse grain concentration on the outlet with 1, 2, 5, and 10 cycles, and $J_{\bar{C}}(C)$ are 0.9882, 0.9779, 0.9404 and 0.8413, respectively.

Mapping method provides an approximate way to obtain the mixing performance of mixer with spatial periodic layout by multiplying the single cycle mapping matrix in corresponding times. Figure 8 shows the evolution of mixing performance of micromixers with different cycles where the single cycle is the layout in Figure 5. Due to the Equation (4) is only valid for situations where fluid agitation is not obvious, we used Equation (3) to measure the mixing performance. One can see that the mixing effect is enhanced as the number of cycle increases. The effect of numerical diffusion becomes apparent, when the multiplying times of mapping matrix increase. However, a mutual comparison is still possible and valuable.

**Figure 9:**
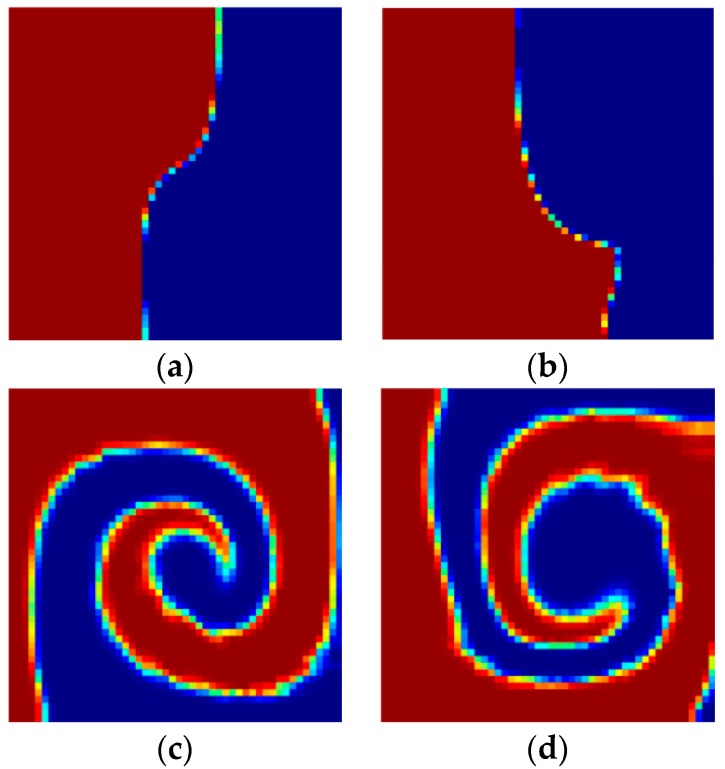
(**a**,**c**) are the distribution of coarse grain concentration on the outlet with 1 and 10 cycles shown in Figure 8a and d, and $J_{\bar{C}}(C)$ are 0.9882 and 0.8413; (**b**,**d**) are the distribution of coarse grain concentration on the outlet of SHM (Figure 10) with 1 and 10 cycles, and $J_{\bar{C}}(C)$ are 0.9883 and 0.8446.

**Figure 10:**
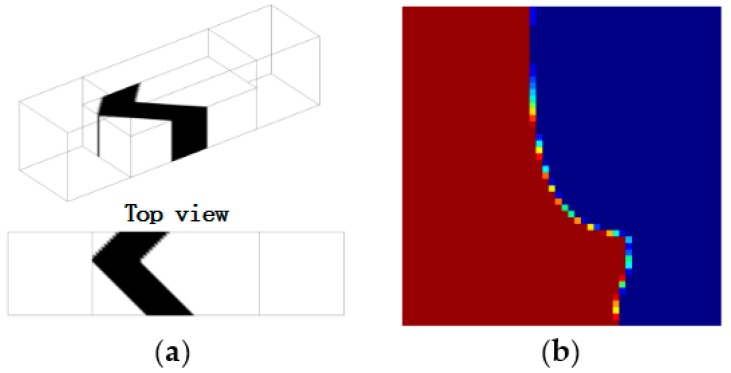
(**a**) Layout of the SHM in the design domain as shown in Figure 4, where *n* = 2, *Re* = 2.5, and the mixing measurement $J_{\bar{C}}(C)$ = 0.9883; (**b**) the distribution of coarse grain concentration on the outlet. (the SHM has only one groove and is same volume as the micromixer shown in Figure 5).

The CPU time in one optimization iteration step is 500 s for the case that design domain is shown in Figure 4, with *n* = 2 and *Re* = 5. Therefore, designing a multi-period structure directly means having a longer computational domain and more CPU time. In contrast, the mapping method can obtain multi-period mixing performance easily by simply multiplying the single cycle matrix in corresponding times (Figure 8). Figure 9 shows the mixing performance of the micromixer that is shown in Figure 5 and SHM shown in Figure 10, which has only one groove and same volume as the micromixer shown in Figure 5. Therefore, there are full of room to adjust the expression of objective in the optimization model (Equation (15)) proposed in this paper. Whereas, the derivation of adjoint sensitivity is unchanged in the case that the chosen objective function is differentiable for the design variable. The whole optimization procedure is valid for immiscible mixer design, as illustrated in this paper.

## 5. Conclusions

In this paper, a novel method is used to design the layout of the passive micromixers of immiscible fluid has been proposed based on the topology optimization of fluidic flows. The layout of the passive micromixers is determined by solving a topology optimization problem to minimize the mixing measurement. Additionally, the detailed mixing performance is obtained by using mapping method in Lagrangian description without consideration of the diffusion. The mapping method used in this paper is a rough approximated method. However, it can produce results that are in good agreement with the conventional numerical simulation method of up to 10 cycles. The discrepancies may increase with the using of more cycles. The design variable is set to represent the impermeability distribution of the artificial porous medium in the design domain. Based on the adjoint analysis of the topology optimization problem, the design variable is evolved to derive impermeability distribution of the artificial porous medium with low and high levels, which, respectively, correspond to the solid and fluid phases in the micromixer. The manufacturability of the obtained layout is ensured by the manufacturing constraint. Numerical results demonstrated the validity of the proposed method. In addition, this method can be extended to design micromixers with multi-layer structure and active micromixers. These will be investigated in our future work.

## Figures and Tables

**Table 1:** Procedure of the iterative approach for solving the topology optimization problem.

Give the initial value of the design variable *γ*; Solve the Navier-Stokes equations and backward particle tracing equation by the finite element method; Solve the adjoint equation; Compute the adjoint derivative and the corresponding objective and constraint values; Update the design variable by method of moving asymptotes (MMA); Check for convergence; if the stopping conditions are not satisfied, go to 2; and Post-processing

## Appendix A

Transfer the constraint model in Equation (15) into unconstrained format by Lagrangian multiplier method:

(A1) $$\begin{array}{cc} L= & J+a{\left(u,\lambda_{u}\right)}_{\Omega }+\rho {\left((u\cdot \nabla )u,\lambda_{u}\right)}_{\Omega }+{\left(\nabla p,\lambda_{u}\right)}_{\Omega }+{\left(\alpha u,\lambda_{u}\right)}_{\Omega }\\ & -{\left(g,\lambda_{u}\right)}_{\Gamma_{N}}+{\left(u-u_{0},\lambda_{u}\right)}_{\Gamma_{D}}+{\left(-\nabla \cdot u,\lambda_{p}\right)}_{\Omega }+{\left(C-\Phi (y,z)C_{0},\lambda_{C}\right)}_{\Gamma_{N}}\\ & +{\left(C-C_{0},\lambda_{C}\right)}_{\Gamma_{D}}+{\left(X-X_{0},\lambda_{X}\right)}_{\Gamma_{D}}+{\left(X_{i}-X_{0}-\sum_{i=0}^{n}\left(\int_{x_{i}+\Delta x}^{x_{i}}\frac{u}{u^{T}I_{u}}dX\right),\lambda_{X}\right)}_{\Gamma_{N}} \end{array}$$

where $a{\left(u,\lambda_{u}\right)}_{\Omega }=\int_{\Omega }\eta \nabla u:\nabla \lambda_{u}d\Omega$; ${\left(*,*\right)}_{\Omega }$ and ${\left(*,*\right)}_{\Gamma }$ is the inner product on the computational domain and boundary; *λ*_u_, *λ_p_*, *λ_C_*, and *λ_X_* are the adjoint variables of the velocity field, the pressure field, the coarse grain density on the cross outlet section and the coordinate after particle tracing, respectively. *λ*_u_ and *λ_C_* are vectors as well as *λ_p_* is scalar. *λ_X_* = (*λ_x_ λ_y_ λ_z_*)^T^. The variation of the main function Equation (A1) is:

(A2) $$\delta L=\frac{\partial L}{\partial u}\delta u+\frac{\partial L}{\partial p}\delta p+\frac{\partial L}{\partial X_{i}}\delta X_{i}+\frac{\partial L}{\partial C}\delta C+\frac{\partial L}{\partial \gamma }\delta \gamma$$

According to the Karush-Kuhn-Tucker conditions for the partial differential equation constrained optimization problem, the optimization model in Equation (15) can be solved by solving the following adjoint equations:

(A3) $$\frac{\partial L}{\partial u}\delta u=0$$

(A4) $$\frac{\partial L}{\partial p}\delta p=0$$

(A5) $$\frac{\partial L}{\partial X_{i}}\delta X_{i}=0$$

(A6) $$\frac{\partial L}{\partial X_{i}}\delta X_{i}=0$$

For the Equation (A3):

(A7) $$\begin{array}{cc} \frac{\partial L}{\partial u}\delta u & =a{\left(\eta \delta u,\lambda_{u}\right)}_{\Omega }+\rho \delta {\left((u\cdot \nabla )u,\lambda_{u}\right)}_{\Omega }+{\left(\alpha \delta u,\lambda_{u}\right)}_{\Omega }-{\left(\nabla \cdot \delta u,\lambda_{p}\right)}_{\Omega }\\ & +{\left(\delta u,\lambda_{u}\right)}_{\Gamma_{D}}-{\left(\int_{x_{0}+\Delta x}^{x_{0}}\left(\frac{1}{u^{T}I_{u}}I-\frac{u}{{\left(u^{T}I_{u}\right)}^{2}}I_{u}^{T}\right)dx,\lambda_{X}\right)}_{\Gamma_{N}} \end{array}$$

where:

(A8) $$a{\left(\eta \delta u,\lambda_{u}\right)}_{\Omega }=-{\left(\eta \Delta \lambda_{u},\delta u\right)}_{\Omega }+{\left(\eta (\nabla \lambda_{u}+{\left(\nabla \lambda_{u}\right)}^{T})\cdot n,\delta u\right)}_{\Gamma_{N}}$$

(A9) $$\delta {\left((u\cdot \nabla )u,\lambda_{u}\right)}_{\Omega }=-{\left((u\cdot \nabla )\lambda_{u},\delta u\right)}_{\Omega }+{\left(\nabla u\cdot \lambda_{u},\delta u\right)}_{\Omega }+{\left((u\cdot n)\lambda_{u},\delta u\right)}_{\Gamma_{N}}$$

(A10) $${\left(\nabla \cdot \delta u,\lambda_{p}\right)}_{\Omega }=-{\left(\nabla \lambda_{p},\delta u\right)}_{\Omega }+{\left(\lambda_{p}n,\delta u\right)}_{\Gamma_{N}}$$

By inserting Equations (A8)–(A10) into Equation (A7), one can obtain:

(A11) $$\begin{array}{cc} \frac{\partial L}{\partial u}\delta u= & \int_{\Omega }\left[-\eta \Delta \lambda_{u}-\rho (u\cdot \nabla )\lambda_{u}+\rho (\nabla u)\cdot \lambda_{u}+\nabla \lambda_{p}+\alpha \lambda_{u}\right]\cdot \delta ud\Omega +\int_{\Gamma_{D}}\left[\lambda_{u}\right]\cdot \delta ud\Gamma \\ & +\int_{\Gamma_{N}}\left[(\eta (\nabla \lambda_{u}+{\left(\nabla \lambda_{u}\right)}^{T}-\lambda_{p}I)\cdot n+\rho (u\cdot n)\lambda_{u}-\sum_{i=0}^{n}\left(\int_{x_{0}+\Delta x}^{x_{0}}\left(\frac{1}{u^{T}I_{u}}I-\frac{u}{{\left(u^{T}I_{u}\right)}^{2}}I_{u}^{T}\right)dX\right)\lambda_{X}\right]\cdot \delta ud\Gamma \\ & =0 \end{array}$$

For the Equation (A4):

(A12) $$\frac{\partial L}{\partial p}\delta p=\int_{\Omega }\left[-\nabla \cdot \lambda_{u}\right]\cdot \delta pd\Omega =0$$

For the Equation (A5):

(A13) $$\frac{\partial L}{\partial X_{i}}\delta X_{i}=\int_{\Gamma_{N}}\left[\lambda_{X}-\frac{\partial \Phi }{\partial X_{i}}\lambda_{C}\right]\cdot \delta X_{i}d\Gamma +\int_{\Gamma_{D}}\left[\lambda_{X}\right]\cdot \delta X_{i}d\Gamma =0$$

For the Equation (A6):

(A14) $$\frac{\partial L}{\partial C}\delta C=\int_{\Gamma_{N}}\left[\frac{-2N(C-C^{0})}{{\left[N+\sum_{i=1}^{N}{\left(C_{i}-C_{i}^{0}\right)}^{2}\right]}^{2}}+\lambda_{C}\right]\cdot \delta Cd\Gamma +\int_{\Gamma_{D}}\left[\lambda_{C}\right]\cdot \delta Cd\Gamma =0$$

Finally, the adjoint equation can be obtained as:

(A15) $$\left\{ \begin{array}{l} -\eta \Delta \lambda_{u}-\rho (u\cdot \nabla )\lambda_{u}+\rho (\nabla u)\cdot \lambda_{u}+\nabla \lambda_{p}=-\alpha \lambda_{u}\;\mathrm{in}\;\Omega \\ -\nabla \cdot \lambda_{u}=0\;\mathrm{in}\;\Omega \\ \lambda_{u}=0\;\mathrm{on}\;\Gamma_{D}\\ {}[\eta (\nabla \lambda_{u}+{\left(\nabla \lambda_{u}\right)}^{T})-\lambda_{p}I]\cdot n=-\rho (u\cdot n)\lambda_{u}+\sum_{i=0}^{n}\left(\int_{x_{0}+\Delta x}^{x_{0}}\left(\frac{1}{u^{T}I_{u}}I-\frac{u}{{\left(u^{T}I_{u}\right)}^{2}}I_{u}^{T}\right)dX\right)\lambda_{X}\;\mathrm{on}\;\Gamma_{N}\\ \lambda_{X}=\frac{\partial \Phi }{\partial X_{i}}\lambda_{C}\;\mathrm{on}\;\Gamma_{N}\\ \lambda_{X}=0\;\mathrm{on}\;\Gamma_{D}\\ \lambda_{C}=0\;\mathrm{on}\;\Gamma_{D}\\ \lambda_{c}=-\frac{-2N(C-C^{0})}{{\left[N+\sum_{i=1}^{N}{\left(C_{i}-C_{i}^{0}\right)}^{2}\right]}^{2}}\;\mathrm{on}\;\Gamma_{N} \end{array}\right.$$

According to the Equations (A3)–(A6) into the variation Equation (A2) of main function, one can obtain:

(A16) $$\delta L=\frac{\partial L}{\partial \gamma }\delta \gamma =\int_{\Omega }\left[\frac{\partial \alpha }{\partial \gamma }u\cdot \lambda_{u}\right]\delta \gamma d\Omega$$

Then the adjoint derivative of the optimization model in Equation (15) is:

(A17) $$\frac{DL}{D\gamma }|{}_{\Omega }=\frac{\partial \alpha }{\partial \gamma }u\cdot \lambda_{u}$$
